# The dynamics of smoking-related disturbed methylation: a two time-point study of methylation change in smokers, non-smokers and former smokers

**DOI:** 10.1186/s12864-017-4198-0

**Published:** 2017-10-18

**Authors:** Rory Wilson, Simone Wahl, Liliane Pfeiffer, Cavin K. Ward-Caviness, Sonja Kunze, Anja Kretschmer, Eva Reischl, Annette Peters, Christian Gieger, Melanie Waldenberger

**Affiliations:** 1 Research Unit of Molecular Epidemiology, Helmholtz Zentrum München, German Research Center for Environmental Health, D-85764 Neuherberg, Bavaria Germany; 2 Institute of Epidemiology II, Helmholtz Zentrum München, German Research Center for Environmental Health, D-85764 Neuherberg, Bavaria Germany; 3 German Center for Diabetes Research (DZD e.V.), München-Neuherberg, Bavaria Germany; 4 Environmental Public Health Division, US Environmental Protection Agency, Chapel Hill, NC 27514 USA; 5 DZHK (German Centre for Cardiovascular Research), partner site Munich Heart Alliance, Munich, Bavaria Germany; 6 Helmholtz Zentrum München, Deutsches Forschungszentrum für Gesundheit und Umwelt (GmbH), Research Unit Molecular Epidemiology (AME), Ingolstädter Landstr. 1, D-85764 Neuherberg, Germany

**Keywords:** DNA methylation, Smoking, Longitudinal study, Epigenetics, Tobacco, Cigarettes

## Abstract

**Background:**

The evidence for epigenome-wide associations between smoking and DNA methylation continues to grow through cross-sectional studies. However, few large-scale investigations have explored the associations using observations for individuals at multiple time-points. Here, through the use of the Illumina 450K BeadChip and data collected at two time-points separated by approximately 7 years, we investigate changes in methylation over time associated with quitting smoking or remaining a former smoker, and those associated with continued smoking.

**Results:**

Our results indicate that after quitting smoking the most rapid reversion of altered methylation occurs within the first two decades, with reversion rates related to the initial differences in methylation. For 52 CpG sites, the change in methylation from baseline to follow-up is significantly different for former smokers relative to the change for never smokers (lowest *p*-value 3.61 x 10^-39^ for cg26703534, gene *AHRR*). Most of these sites’ respective regions have been previously implicated in smoking-associated diseases. Despite the early rapid change, dynamism of methylation appears greater in former smokers vs never smokers even four decades after cessation. Furthermore, our study reveals the heterogeneous effect of continued smoking: the methylation levels of some loci further diverge between smokers and non-smokers, while others re-approach. Though intensity of smoking habit appears more significant than duration, results remain inconclusive.

**Conclusions:**

This study improves the understanding of the dynamic link between cigarette smoking and methylation, revealing the continued fluctuation of methylation levels decades after smoking cessation and demonstrating that continuing smoking can have an array of effects. The results can facilitate insights into the molecular mechanisms behind smoking-induced disturbed methylation, improving the possibility for development of biomarkers of past smoking behavior and increasing the understanding of the molecular path from exposure to disease.

**Electronic supplementary material:**

The online version of this article (10.1186/s12864-017-4198-0) contains supplementary material, which is available to authorized users.

## Background

Tobacco use causes up to 6 million deaths per year [1] – 10% of adult deaths [2] – primarily through cancer, chronic obstructive pulmonary disease and cardiovascular disease [3]. The link between smoking and adverse health outcomes is well established but the precise causal molecular and cellular mechanisms are still under investigation [4–6]. Epigenetic modifications, such as DNA methylation at cytosine-guanine dinucleotides (CpGs), are thought to be potential mediators in the course from exposure to disease.

The link between smoking and methylation is receiving growing attention [7], with numerous recent cross-sectional studies revealing epigenome-wide associations [8–12]. The most recent, comprehensive analyses include a systematic review of 17 studies [13], and a meta-analysis of 16 cohorts [12], the latter revealing 2623 CpGs sites significantly associated with smoking behavior at a strict multiple testing threshold. However, longitudinal studies – here defined as those examining methylation and smoking habits in individuals at two or more time-points – are scarce. These studies are an important next step in the understanding of the mechanisms of methylation [14]. To our knowledge, there have been only three reports investigating smoking and methylation with repeated measures: two candidate locus studies, one focusing on a small group of young women [15] and another on a small group of individuals attempting to quit smoking [16]; and a family-based study focused on maternal smoking [17]. Studies which cover larger sample sizes and different life stages are needed to better understand the effect of smoking on methylation.

A key question in the field of smoking-mediated methylation is whether quitting smoking allows differentially methylated CpG sites to return to levels found in individuals who have never smoked. Previous evaluations of the effect of time since smoking cessation have revealed CpG sites that could be classified as reversible and sites that could be classified as persistently differentially methylated [8, 18–20]. Reversible sites are those that are differentially methylated between smokers and non-smokers but with the difference disappearing some time after cessation of smoking. Persistently differentially methylated sites remain differentially methylated, perhaps indefinitely; this persistence has been observed even up to 35 years after cessation [20]. However, there is again a lack of longitudinal studies with regard to reversion of methylation levels; this lack impedes the identification of potential long-term biomarkers of smoking [11, 21], and hinders insights into the increased risk for disease faced by former smokers decades after cessation [22, 23].

The focus of our investigation is the linking of exposure – current and prior smoking behavior – to changes in DNA methylation at CpG sites over time, i.e. dynamism in methylation. Our previous study, a cross-sectional epigenome-wide association study (EWAS), compared the methylation of current, former and never smokers at approximately 450,000 CpG sites, and revealed an extensive effect of smoking across the methylome [8]. Here, we extend this investigation to include an earlier time-point for the same cohort, thus gaining information on changes in smoking habits, health characteristics and DNA methylation. Through a longitudinal site-by-site analysis, our goals were to (i) examine changes in methylation over time associated with quitting smoking or remaining a former smoker; and (ii) examine the effects of continued smoking on changes in DNA methylation, including the effect of intensity of smoking habit.

## Methods

### Study population

Our study population consisted of participants from the KORA (Kooperative Gesundheitsforschung in der Region Augsburg) study [24], which has been collecting clinical and genetic data from the general population in the region of Augsburg, Germany for more than 20 years. The cohort investigated in this paper is the S4 study, carried out in 1999–2001 (baseline). The follow-up (F4) took place in 2006–2008. At both assessments, participants completed a lifestyle questionnaire, including details on smoking habits, and underwent standardized examinations with blood samples taken, as described elsewhere [24, 25].

Individuals who were either regular or occasional (self-declared as 1 cigarette per day or less) smokers at the time of the interview were classified as current smokers (CS); those who had never smoked were classified as never smokers (NS); and those who had previously been smokers but were no longer at the time of the interview were former smokers (FS).

Since the analyses involve longitudinal data, smoking status of an individual may change between the time-points. The above-noted abbreviations separated by a dash indicate smoking statuses at baseline and follow-up interviews. For example, CS-FS refers to the category of individuals who were smokers at the time of baseline and former smokers at the time of follow-up.

We calculated duration of smoking habit as the difference between age of smoking initiation and age at interview for the current smokers, and age of cessation for the former smokers. An individual’s time since quitting smoking (TSQ) was calculated as the difference between the age of cessation and age at the time of the interview. Intensity of smoking is given as the average number of cigarettes smoked per day at the time of the interview; occasional smokers were assumed to smoke 0.5 cigarettes per day.

Of the 4261 baseline subjects, 3080 participated in the follow-up. Of these, 1561 and 1802 had methylation measurements, respectively. After methylation quality control (see below), 1535 samples remained in S4 and 1727 remained in F4. 91 observations were lacking data for one or more of the covariates and were excluded. 158 FS observations were eliminated due to lack of TSQ data, inconsistent TSQ information given at S4 and F4 (TSQ varying by greater than 20% and more than 5 years), or re-starting and re-quitting between the times of S4 and F4. This left a total 1344 individuals, each with two observations.

### Microarray data acquisition

DNA methylation was measured in the whole blood of the participants using the Infinium HumanMethylation450K BeadChip. The bisulfite conversion and genome-wide methylation assessment were performed as previously described [8]. All presented gene and position annotations are based on the HumanMethylation450 v1.2 manifest file.

### Methylation data preprocessing

Normalization of the methylation data was conducted following the CPACOR pipeline [26], beginning with exclusion of 65 single-nucleotide polymorphism markers and background correction using the R package minfi [27]. Probes were set to NA if the detection *p*-value ≥0.01 or number of beads ≤3. Samples were excluded if the detection rate was ≤0.95. Quantile normalization was then performed on the signal intensities.

The methylation of a given cytosine was first calculated as a β-value, the ratio of the methylated signal intensity to the sum of the methylated and unmethylated signal intensities. Due to the [0,1] boundedness of the β-values, they were transformed to M-values using the binary logit transformation, M-value = log_2_(β/(1- β)), for all analyses in this study, except where noted.

Following normalization, a per-CpG-site detection rate of 95% was applied to the baseline and follow-up studies separately. CpG sites with a detection rate below 95% for either baseline or follow-up were excluded from all analyses, resulting in a reduction from 485,512 to 459,472 sites; after exclusion of sites from the sex chromosomes the final number analyzed was 449,102.

To reduce possible impact of non-biological effects, specifically those differing between the experiments for the S4 and F4 samples, and 86 individuals from S4 processed separately, we adjusted the methylation M-values for technical effects prior to analysis. In detail, principal component analysis was performed on the intensities of all (non-negative, autosomal) control probes after background correction. We then modeled the methylation M-values of each CpG site across all samples as a function of the first 20 principal components, plus a batch indicator designating the *n* = 86 S4 subsample. Residuals of these models were used as “technically adjusted” methylation values for all analyses [26].

To eliminate potential outliers for each CpG site, the residuals of all S4 and F4 individuals from a linear regression model featuring methylation as response and all potential confounders (see below) as covariates were examined. Outliers were defined as those values more than 5 standard deviations from the mean. Up to 5 outliers were removed per CpG site, 5 being chosen to maintain sample size.

### Statistical models and methods

Our analysis incorporates cross-sectional and longitudinal models. For the cross-sectional models, the baseline data is used alone. For all longitudinal models, the baseline and follow-up data are used.

### Confounding

For all regression models, sex, alcohol consumption (g/day), body mass index, white blood cell count and estimated white blood cell proportions (monocytes, B cells, natural killer cells, CD4 T cells and CD8 T cells, estimated using the method of [28]) at the time of the examination were included as covariates to address potential confounding. For the cross-sectional models, age was included, while for the longitudinal models, age at baseline was included for each observation along with a time passed variable (0 for baseline, time difference between baseline and follow-up interviews for follow-up observations) to account for the longitudinal nature of the data; see model description below.

### Confounder residualization for methylation beta value analysis

In some analysis we examine methylation beta values rather than coefficients of regression models. In these cases, to address potential confounding, we perform another stage of residualization, similar that described to remove technical effects. For each CpG site, we conduct a linear regression model with the technically adjusted beta values as outcome and all covariates as independent variables. The residuals of these models are our final ‘methylation’ values for the analyses relying on beta values rather than regression coefficients.

### Cross-sectional analysis: Epigenome-wide association analysis at baseline

We firstly conducted an EWAS on the baseline data to investigate which CpG sites were differentially methylated between CS (*N* = 280) and NS (*N* = 615). For each CpG site, the technically adjusted M-values were used as outcome in a linear regression model with smoking status (NS or CS) as the explanatory variable and covariates as above. A total of 449,102 CpG sites were tested; thus a CpG site was considered significant if the coefficient of smoking status for its model had a *p*-value below a Bonferroni-corrected threshold of ~1.1 × 10^−7^.

The EWAS indicated 590 CpG sites were associated with smoking behavior, and these sites were carried forward to all other analyses. From this point results were considered significant at a Bonferroni-corrected P of 8.47 × 10^−5^.

### Longitudinal analyses: Baseline to follow-up

The longitudinal analyses involve data from the two time-points, baseline and follow-up. We use models adapted from Richmond et al. [17]. For each CpG site, we used a linear mixed model with random intercept of individual and the following structure for individual *i* at time point *j* (*j* = 1 at baseline, *j* = 2 at follow-up) to model the methylation:

$$ {\displaystyle \begin{array}{c}{meth}_{ij}={\beta}_0+{\beta}_c smoking\kern0.5em category\kern0.5em +{\beta}_1 age\kern0.5em at\kern0.5em baseline\\ {}+\kern0.5em {\beta}_t time\kern0.5em passed\kern0.5em since\kern0.5em baseline\kern0.5em interview\kern0.5em +\kern0.5em {\beta}_L smoking\kern0.5em category\\ {}\times \kern0.5em \left( time\kern0.5em passed\kern0.5em since\kern0.5em baseline\kern0.5em interview\right)+\kern0.5em {confounders}_{ij}+{\mu}_{0i}+{\varepsilon}_{ij}\end{array}} $$

$$ {\mu}_{0i}\sim N\left(0,{\sigma}_{\mu}^2\right) $$

$$ {\varepsilon}_{ij}\sim N\left(0,{\sigma}_{\varepsilon}^2\right), $$

where the smoking category depends on the model in question (see below), and the time passed since baseline is 0 at *j* = 1 and the time in years since the baseline interview for *j* = 2. β_C_ thus can be interpreted as the expected methylation difference between an individual of the smoking category in question and the reference category at baseline, given equality of all other covariates; β_t_ gives the expected change in methylation per year from baseline to follow-up for the reference category; β_L_ gives the expected difference in change in methylation per year from baseline to follow-up for the smoking category in question vs the reference category, given equality of the other covariates. We refer to β_C_ as the *cross-sectional* coefficient or effect and to β_L_ as the *longitudinal* coefficient or effect.

For each analysis, a “significant site” refers to a site that is significant for the longitudinal effect.

### Quitting smoking or remaining a former smoker

To investigate the effect of quitting smoking or remaining a former smoker, we examined the change in methylation over the time from baseline to follow-up for FS (CS-FS and FS-FS) in comparison to the change for the NS-NS individuals. To also capture those individuals who quit smoking between the two time-points, we calculated an individual’s TSQ as the number of years since smoking cessation at the time of the follow-up interview. Using break points chosen to match sample sizes across categories, we categorized TSQ, which ranged from >0 to 70 years, into 7 categories (TSQ_L_) (see Additional file 1: Table S1). These were compared to reference NS-NS (*n* = 614) using a longitudinal model as described above, with 8 total smoking categories (the 7 TSQ_L_ categories and the reference NS-NS individuals). Further adjustment for TSQ within each category was not performed.

The cross-sectional effect of this model indicates the baseline difference between the methylation levels of the given TSQ_L_ category and the NS-NS individuals. The longitudinal effect indicates the rate of change of methylation per year between baseline and follow-up, relative to the rate of change for the NS-NS over the same time period.

### Continuing smoking

To investigate the effect of continued smoking, we compared the change in DNA methylation from baseline to follow-up for those individuals who were CS at both time points (CS-CS, 181 individuals) to the reference NS-NS (614 individuals) using a longitudinal model as described above, with 2 total smoking categories (CS-CS individuals and the reference NS-NS individuals).

The cross-sectional effect of this model indicates the difference between the methylation levels of the CS-CS category and the individuals of NS-NS at baseline. The longitudinal effect indicates the rate of change of methylation per year between baseline and follow-up for the CS-CS, relative to NS-NS.

All statistical analyses were conducted with R statistical software version 3.3.3 [29], with package lme4 [30] version 1.1–12 and lmerTest version 2.0–32 [31] for the linear mixed models, and figures created using ggplot2 version 2.0.0 [32].

## Results

### Population characteristics

The characteristics for the current smokers (CS), former smokers (FS) and individuals who have never smoked (NS) are given in Table 1. For both time-points, CS and FS were more often male than female, were slightly younger than NS and had heavier alcohol intake. FS tended to have a slightly higher body mass index than CS or NS. Between baseline and follow-up, the number of FS grew, the number of CS fell and the number of NS stayed roughly equal.

**Table 1:** Population characteristics for baseline and follow-up studies. Mean ± standard deviation or N (%)

	Baseline				Follow-up			
	Current smokers	Former smokers	Never smokers	*P*-value*	Current smokers	Former smokers	Never smokers	*P*-value*
N	280	449	615	–	207	523	614	–
Males (%) ^#^	0.57	0.64	0.35	< 2.2e-16	0.55	0.64	0.35	< 2.2e-16
Age (years) ^##^	50.8 ± 7.8	54.7 ± 8.8	55.1 ± 9.0	< 1e-7	57.1 ± 7.0	61.5 ± 8.9	62.2 ± 9.0	< 1e-7
Body mass index (kg/m2) ^##^	27.1 ± 4.5	28.1 ± 4.5	27.6 ± 4.3	< 1e-3	27.2 ± 5.0	28.5 ± 4.9	27.9 ± 4.5	< 0.05
Alcohol consumption (g/day) ^##^	20.2 ± 25.9	20.5 ± 23.1	13.1 ± 18.2	< 1e-7	18.2 ± 24.3	19.1 ± 22.2	12.1 ± 17.1	< 1e-7
Duration of smoking (years) ^##^	31.4 ± 9.3	17.0 ± 10.6	–	< 2.2e-16	36.8 ± 10.6	20.7 ± 12.9	–	< 2.2e-16
Average intensity of smoking (cig/day)	14.9 ± 10.9	–	–	–	12.6 ± 9.0	–	–	–
Time since quitting smoking (years)	–	19.9 ± 10.8	–	–	–	23.1 ± 13.6	–	–

*P-value of test for equality between the groups (current smokers, former smokers, never smokers)

^#^ *P*-value for equality between groups determined by the chi-square test for independence

^##^ *P*-value for equality between groups determined by the Kruskall-Wallace test by ranks

### Cross-sectional analysis: Epigenome-wide association analysis at baseline

A total of 590 CpG sites were found to be associated with smoking status (see Additional file 2: Table S2). For all following analyses, only these 590 significant CpG sites were examined.

### Longitudinal analysis of time since quitting smoking

**Fig. 1:**
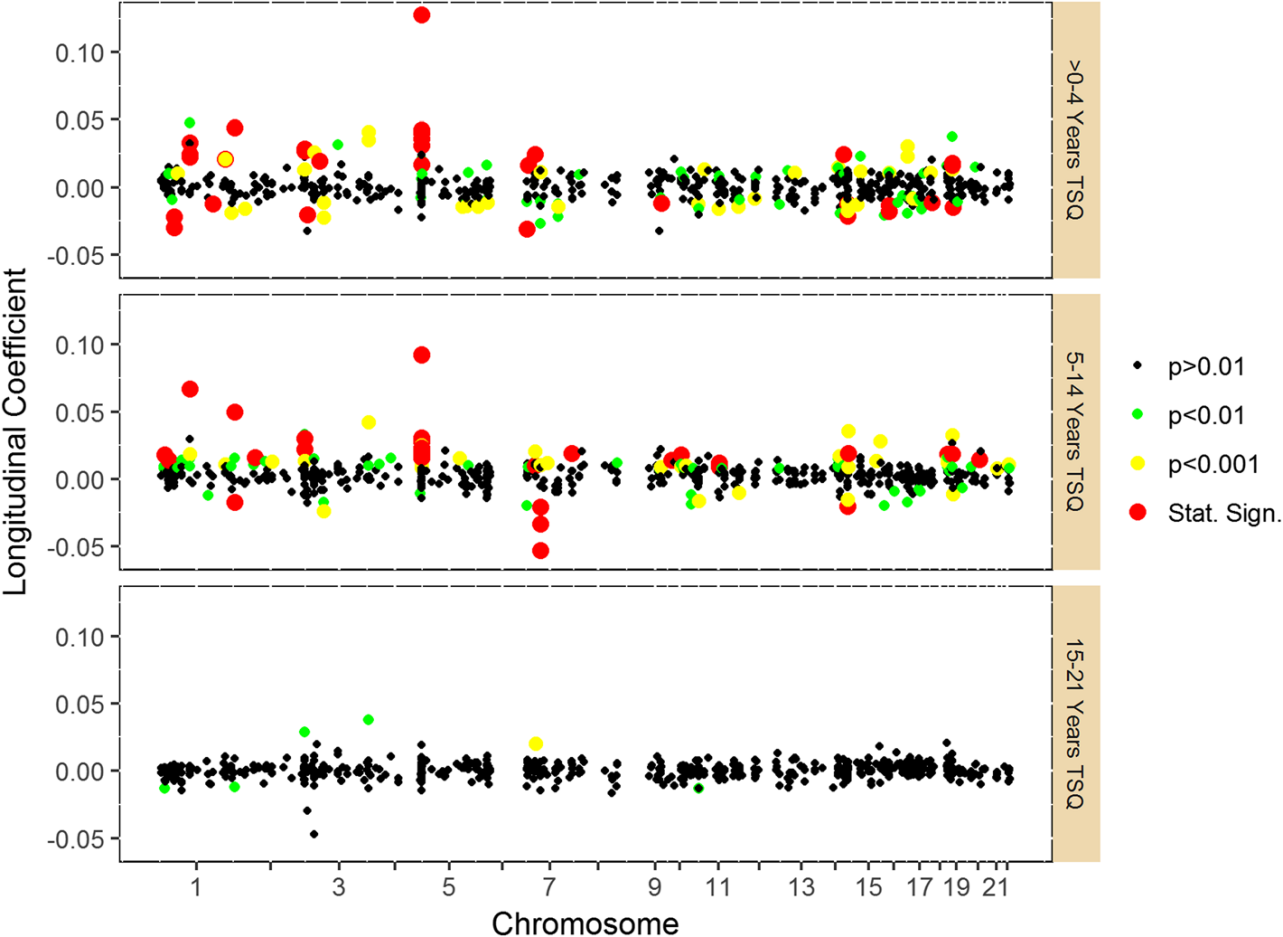
Longitudinal regression coefficients for each CpG site under investigation longitudinally. The three panels display the longitudinal coefficients and coefficient p-values for TSQ_L_ categories 1, 2 and 3, respectively, for each CpG site under investigation. The longitudinal coefficients represent the rates of change of methylation M-value per year relative to never smokers. The results for TSQ_L_ categories 4 through 7 are given in Additional file 3: Figure S1. *Statistically significant: the longitudinal coefficient P falls below the Bonferroni-corrected threshold of 8.47 × 10^−5^

The results, within our analysis framework, indicate that for the FS the greatest alteration in methylation patterns, relative to NS, occurs within the first 14 years after quitting smoking (Fig. 1, Additional file 3: Figure S1). The longitudinal coefficients are largest for the first two TSQ_L_ categories, and are relatively small following this point. For those who quit (>0)-4 years prior to the follow-up exam (TSQ_L_ category 1) 32 CpG sites show a significant differential change in methylation compared to NS-NS. Of these, 10 remain significant for individuals of TSQ_L_ category 2 (5–14 years TSQ) and none were significant for individuals who quit more than 14 years prior to the follow-up examination. Twenty additional sites show differential change in methylation for individuals of TSQ_L_ category 2 without being significant for individuals of category 1. All directions of change for these 52 significant CpG sites are consistent with regard to the general effect of smoking seen in the EWAS for each site, i.e. the effects are of opposite sign, indicating reversion to NS levels. Considering all 590 sites, 82% of sites show consistent direction of effect for TSQ_L_ category 1 (>0–4 years TSQ), 82% for category 2 (5–14 years TSQ), and then falling to 46%, 59%, 49%, 47%, and 45% for the respective remaining categories.

### Biological relevance

Additional file 4: Table S3 details these 52 CpG sites, which together cover 33 different genes or regions, gene *AHRR* the most prominent (8 sites total, lowest overall *p*-value 3.61 x 10^-39^ for cg26703534). Other loci showing multiple significant sites include *GFI1* (4 sites), *MYO1G* (3 sites), 2q37.1 (3 sites), *HIVEP3* (2 sites), 2p25.1 (2 sites) and *ZNF668* (2 sites). Additional file 4: Table S3 also presents the results for all 590 sites.

All regions corresponding to the CpG sites significant in this analysis, and all CpG sites themselves, have previously been identified as being associated with smoking [8, 12, 33–36], and discussed with regard to their biological implications in a number of publications, in particular 2q37.1 [8, 13, 37], *AHRR* [7, 38, 39], *GFI1* [40], *MYO1G* [41] and *F2RL3* [7, 42]. Further, most of these loci have been identified as associated with conditions or diseases also related to smoking. Additional file 5: Table S4 presents a (non-exhaustive) list of the sites identified in this analysis with their loci’s associations with disease, and how these diseases have been previously linked to smoking. As would be expected, the genes identified in this study have been implicated in the smoking-associated conditions osteoporosis (*LRP5*), inflammatory bowel disease (*CPAMD8*, *GRP68*), cognitive disorders (*AVPR1B*, *SYNJ2*), male infertility (*AHRR*), Parkinson’s (*HIVEP3*, *HTRA2*), rheumatoid arthritis (*CD247*), atherosclerosis (*AHRR*) and a wide array of cancers (many genes). Specific CpG sites identified here have been found to be associated with lung cancer (cg05951221 and cg21566642 of 2q37.1, cg05575921 of *AHRR*, cg03636183 of *F2RL3*), atherosclerosis (cg05575921 of *AHRR*), body mass index (cg23576855 of *AHRR*, cg09554443 of *CD247*), and mortality (cg05575921 of *AHRR*).

Further biological insights can be achieved through gene analysis using the WEB-based GEne SeT AnaLysis Toolkit (WebGestalt) [43] (see Additional file 6: Table S5). Using the list of genes annotated to any CpG site significant for any TSQ_L_ category at a nominal *p* < 0.05, we ran overrepresentation enrichment analysis based on the GLAD4U disease functional database [44], the default parameters (5–2000 genes per category, Benjamini-Hochberg multiple-testing correction) and the reference set “illumina_human_methylation_450”. The results indicate leukemia (as well as myeloid leukemia and acute myeloid leukemia) is significantly associated with our list of genes at a false discovery rate (FDR) of 1.11e-3. Using lists of only the genes annotated to CpG sites significant (*p* < 0.05) for TSQ_L_ category 2 or later, TSQ_L_ category 3 or later and TSQ_L_ category 4 or later, we see similar results. The disease category “mouth neoplasms” is borderline significant for most of these analyses as well, achieving its smallest FDR of 2.03e-02 for the list based on TSQ_L_ category 3 or later. The implication of these analyses is that the genes with CpG sites showing change in methylation more dynamic than NS even up to 22–27 years since cessation (TSQ_L_ category 4) are an overrepresentation of genes associated with leukemia (and, to a lesser extent, mouth neoplasms), a disease well known to be more prevalent in smokers.

Using the same parameters, but instead focusing on gene set enrichment analysis (KEGG pathway functional analysis), “pathways in cancer” is found to be significant (17 genes, FDR = 0.0114) if we consider all significant genes from TSQ_L_ category 1 or later. It remains the top pathway considering all genes from TSQ_L_ 2 or later and TSQ_L_ 3 or later as well (14 genes, FDR = 0.10; 12 genes, FDR = 0.11; respectively). These are further indications that, for former smokers, even decades after smoking cessation, dynamic methylation is apparent in genes in cancer-related pathways.

### Methylation dynamics on a finer scale

**Fig. 2:**
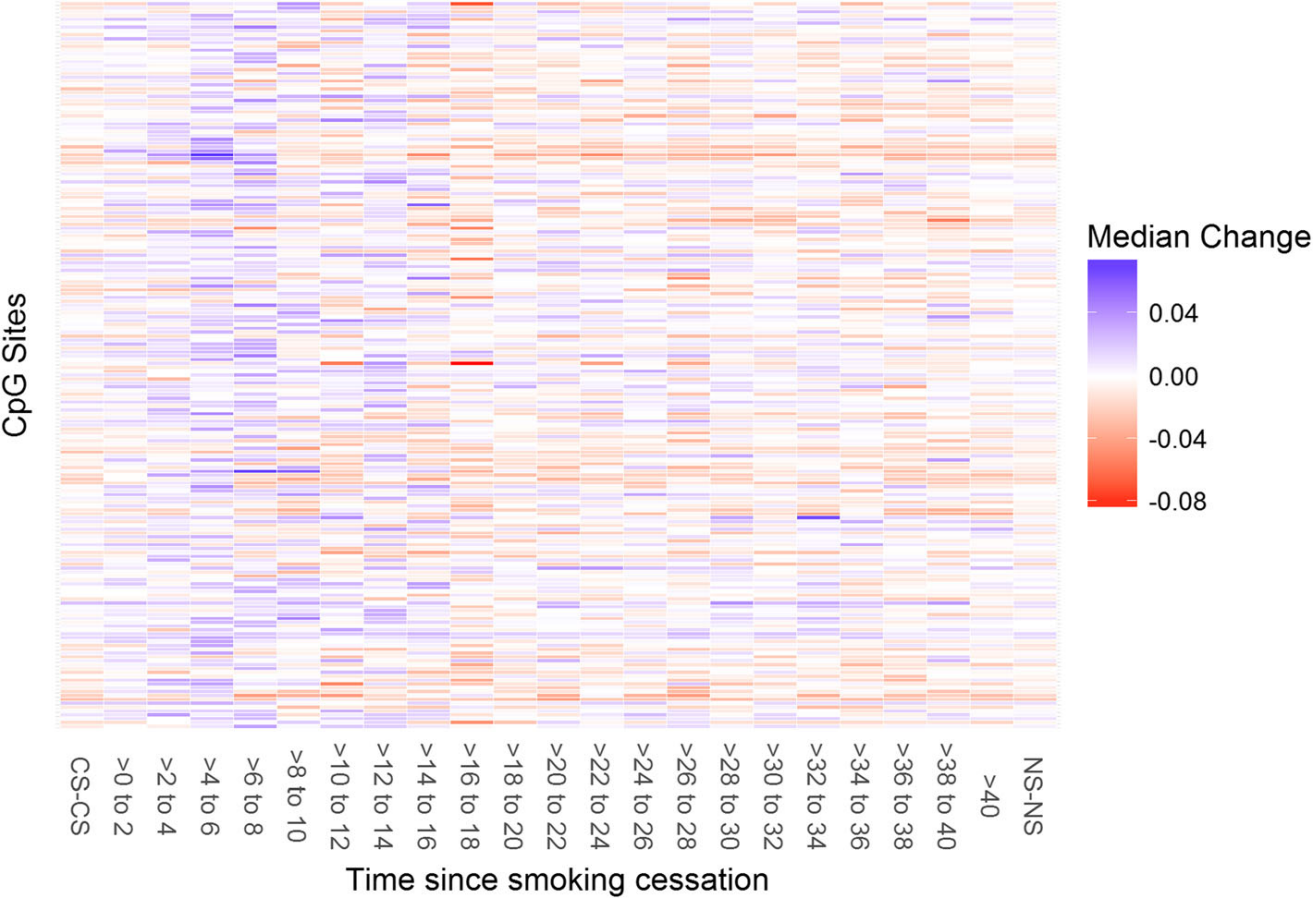
Heat map of median changes in methylation beta value from baseline to follow-up. Displayed are the results for current smokers, former smokers and never smokers. The color indicates direction of change in relation to the effect of smoking as found in the epigenome-wide analysis: red is the same direction, blue is opposite. Presented are only those CpG sites with a median change greater than 0.025 in at least one smoking category. Additional file 7: Figure S2 presents all sites

**Fig. 3:**
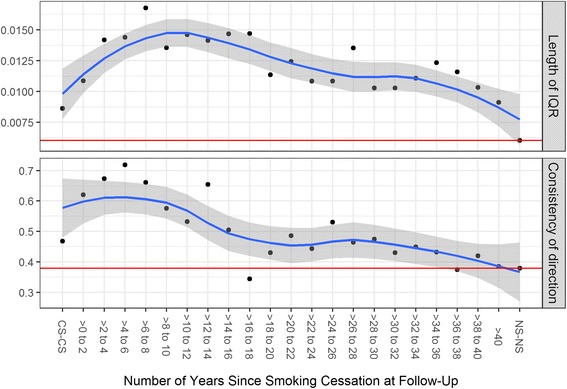
Change in methylation beta values from baseline to follow-up, 2-year intervals. Displayed are the results for current smokers, former smokers and never smokers. Upper panel: gives the length of the interquartile range over all CpG sites of the median change in methylation. A larger interquartile range indicates greater fluctuation in methylation between baseline and follow-up over the 590 CpG sites. Lower panel: gives the proportion of sites with consistent direction of change to the effect of smoking as found in the epigenome-wide analysis, “consistent” defined here as opposite in sign to the baseline effect of smoking. For both panels, the red line indicates the value for the never smoking individuals, the blue line is the smoothing loess curve as defined by the stat_smooth function with default values from the R package ggplot2, and the gray band is its 95% confidence interval. Additional file 8: Figure S3 shows the same analysis but for the original TSQ_L_ categories

To investigate the dynamics of methylation for FS on a finer scale, we calculate the change in (beta value) methylation from baseline to follow-up for each individual for each CpG site, firstly employing confounder residualization, as described in the Statistical Methods. We then divide the FS into TSQ categories of 2 years and, within each category as well as within the NS-NS, calculate each CpG site’s median change in methylation (visualized as heat maps in Fig. 2 and Additional file 7: Figure S2). Examining the results plotted in Fig. 3, we see that the largest dynamism in methylation – here expressed as the size of the interquartile range across all 590 CpG sites of median change in methylation – occurs for individuals between approximately 5 and 18 years TSQ, depending on subjective interpretation. The proportion of CpG sites with consistent direction of change for the median change, i.e. opposite in sign to the EWAS effect of smoking for that site, is given also in Fig. 3, the results indicating a relatively steady decrease over time of CpG sites that have their methylation levels moving towards those of NS. The same analysis, but for the original TSQ_L_ categories, is given in Additional file 8: Figure S3.

### Rate of reversion

**Fig. 4:**
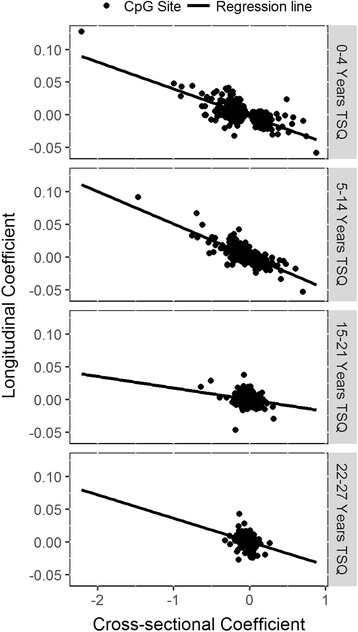
Cross-sectional coefficient vs longitudinal coefficient for each CpG site under investigation longitudinally. The panels display the results for TSQ_L_ category 1, category 2, category 3 and category 4, respectively. The longitudinal coefficients represent the rates of change of methylation M-value per year relative to never smokers. The cross-sectional coefficients represent the baseline difference in methylation M-value compared to never smokers

Of particular interest is the effect of the absolute difference between methylation levels of FS and NS on the rate of change of methylation for FS. In Fig. 4, we plot the cross-sectional coefficients for the first four TSQ_L_ categories vs the respective longitudinal coefficients, for all 590 CpG sites. The absolute change in methylation between baseline and follow-up, corresponding to the longitudinal coefficient, tends to be larger for larger initial absolute differences in methylation, i.e. larger cross-sectional coefficient. For the first two TSQ_L_ categories there is a very high negative correlation between rate of change (longitudinal coefficient) and starting methylation difference (cross-sectional coefficient) over all sites: TSQ_L_ category 1, Spearman’s ρ = −0.65 (*p* < 2.2 × 10^−16^); TSQ_L_ category 2, ρ = −0.69 (*p* < 2.2 × 10^−16^). The absolute correlations are greater if we consider only those sites which show significance in one or more TSQ_L_ categories (Additional file 9: Table S6). The correlations decrease in magnitude for further TSQ_L_ categories, but remain negative.

### Longitudinal effect of continued smoking

Of the 590 CpG sites examined, 14 showed a Bonferroni-corrected significant difference in change in methylation over time (Table 2, see Additional file 10: Table S7 for all results). Of immediate note is that the longitudinal effect is in the opposite direction to the cross-sectional effect for 5 CpG sites: cg05575921 and cg09338136 of *AHRR*, cg05875421 of *GPR68*, cg25512107 of *RPTOR* and cg23079012 at 2p25.1. Additional file 11: Figure S4 presents boxplots of the methylation for both time points, giving visual indication of the heterogeneous effects. An insignificant Spearman ρ of −0.16 (*p* = 0.58) between the longitudinal and cross-sectional coefficients for those sites with significant longitudinal effect in this analysis further highlights this lack of consistency. This correlation lies in stark contrast to those for the TSQ analysis, which were greater in magnitude, of consistent direction, and statistically significant. Of all 590 sites in the continued smoking analysis, only 315 (53%) had the same direction for the cross-sectional effect and the longitudinal effect (with ρ = 0.08, *p* = 0.052).

**Table 2:** Statistically significant results of the analysis of longitudinal effect of continued smoking

CpG site	CHR	Gene or region	Cross-sectional coefficient	Longitudinal coefficient	Longitudinal coefficient *p*-value	Median methylations (CS vs NS) diverging or converging from baseline to follow-up
cg13184736	1	*GNG12*	−0.339	−0.019	1.36E-05	Div.
cg25189904	1	*GNG12*	−0.507	−0.016	1.26E-06	Div.
cg23079012	2	2p25.1	−0.887	0.039	3.82E-09	Con.
cg05575921	5	*AHRR*	−2.575	0.061	7.83E-05	Con.
cg09338136	5	*AHRR*	−0.156	0.011	5.29E-18	Con.
cg06126421	6	6p21.33	−0.697	−0.017	1.23E-08	Con.^a^
cg14753356	6	6p21.33	−0.237	−0.012	7.94E-07	Div.
cg12147622	10	10q22.1	−0.125	−0.012	8.05E-05	Div.
cg05875421	14	*GPR68*	−0.211	0.017	1.23E-05	Con.
cg15022400	15	*TRIM69*	−0.112	−0.012	3.37E-06	Div.
cg23161492	15	*ANPEP*	−0.306	−0.010	5.69E-05	Div.
cg07251887	17	*LOC100130933*	−0.132	−0.011	5.26E-07	Div.
cg25512107	17	*RPTOR*	−0.336	0.037	2.48E-08	Con.
cg15187398	19	*MOBKL2A*	−0.161	−0.010	8.51E-06	Div.

CHR: chromosome of the CpG site; CS: current smokers; NS: never smokers

^a^methylation levels are converging, but the longitudinal and cross-sectional coefficients are of the same sign

Legend: Presented are the 14 CpG sites significant for the longitudinal coefficient in the continued smoking analysis. The longitudinal coefficient represents the difference in rate of change of methylation M-value between baseline and follow-up for the individuals who were smokers at both time points relative to the individuals who were never smokers at both time points. Also presented is the cross-sectional coefficient, which represents the difference in methylation at baseline for the same two groups. A CpG site is labelled as “diverging” if the median methylations between the two groups separate further from baseline to follow-up; a CpG site is labelled as “converging” if the median methylations between the two groups approach one another from baseline to follow-up

### Intensity vs duration of smoking habit

To explain this result one can examine the possibility that intensity of smoking habit is the predominant factor rather than duration of smoking habit for the sites in question. If so, the fact that in our study the mean intensity for CS-CS individuals decreased from 16.5 (sd = 11.4) to 13.3 cigarettes/day (sd = 8.9) between baseline and follow-up may provide clarity. To compare the effects of current intensity of smoking (average number of cigarettes smoked per day at the time of the interview) vs duration of smoking habit, we ran a linear mixed model of CS individuals (*N* = 181 × 2 time-points = 362 data points), with methylation (technically adjusted M-values) as outcome, intensity and duration as independent variables and individual as random intercept, adjusted for the potential confounders detailed in the Statistical Methods. Intensity was significantly associated with methylation at 32 CpG sites (annotated to 24 genes or regions), while duration of smoking was associated with methylation at only 1 (Table 3, see Additional file 12: Table S8 for all sites). The directions of effect for increased intensity were consistent with the cross-sectional effect for smoking found in the EWAS for each of the 32 sites, i.e. the same direction; likewise for the significant hit for duration. However of the 5 CpG sites showing significant longitudinal effect in the continued smoking analysis above with opposite cross-sectional and longitudinal signs, only cg05575921 (*AHRR*) showed significance for smoking intensity.

**Table 3:** Statistically significant results for the model incorporating both intensity and duration of smoking

CpG site	CHR	Gene or position	Intensity of smoking coefficient	Duration of smoking coefficient	Intensity of smoking coefficient *p*-value	Duration of smoking coefficient *p*-value	EWAS coefficient for CpG site
cg09935388	1	*GFI1*	−1.15E-02	−1.85E-02	9.21E-06	5.83E-03	−5.18E-01
cg08709672	1	*AVPR1B*	−5.78E-03	4.31E-04	1.01E-06	8.49E-01	−1.98E-01
cg03329539	2	2q37.1	−6.49E-03	−2.87E-03	3.11E-05	2.63E-01	−3.24E-01
cg05951221	2	2q37.1	−1.08E-02	−5.97E-03	3.75E-09	9.87E-02	−6.68E-01
cg21566642	2	2q37.1	−1.23E-02	−7.27E-03	5.54E-07	1.35E-01	−9.99E-01
cg01940273	2	2q37.1	−8.37E-03	−4.38E-03	2.28E-08	1.58E-01	−6.16E-01
cg00501876	3	*CSRNP1*	−5.33E-03	−1.89E-03	5.24E-06	3.29E-01	−1.57E-01
cg19859270	3	*GPR15*	−7.45E-03	−7.58E-03	4.19E-05	6.36E-03	−3.02E-01
cg02657160	3	*CPOX*	−7.25E-03	−7.50E-03	2.45E-05	4.66E-03	−1.79E-01
cg23576855	5	*AHRR*	−1.59E-02	−1.16E-02	9.81E-08	2.47E-01	−1.00E + 00
cg05575921	5	*AHRR*	−4.32E-02	−1.78E-02	< 2e-16	8.51E-02	−2.38E + 00
cg26703534	5	*AHRR*	−6.54E-03	−1.34E-03	2.61E-07	5.70E-01	−3.39E-01
cg25648203	5	*AHRR*	−1.04E-02	−6.48E-03	1.75E-07	1.02E-01	−4.13E-01
cg21161138	5	*AHRR*	−1.09E-02	−7.39E-03	8.01E-09	3.69E-02	−4.72E-01
cg02451831	7	*KIAA0087*	−6.99E-03	−2.85E-03	3.05E-05	3.34E-01	−1.74E-01
cg10750182	10	*C10orf105*	−3.78E-03	−2.89E-03	4.20E-05	5.33E-02	−1.27E-01
cg02743070	10	*ZMIZ1*	−4.56E-03	−4.68E-04	2.30E-05	7.80E-01	−8.71E-02
cg03450842	10	*ZMIZ1*	−4.54E-03	−2.07E-03	5.93E-05	3.01E-01	−1.52E-01
cg21611682	11	*LRP5*	−4.91E-03	−1.35E-03	6.35E-06	5.14E-01	−1.89E-01
cg11660018	11	*PRSS23*	−5.21E-03	−4.93E-03	5.94E-05	7.83E-02	−2.33E-01
cg13525276	14	*TSHR*	8.15E-03	4.73E-03	4.76E-05	1.68E-01	1.88E-01
cg18625627	14	*TSHR*	8.46E-03	4.39E-03	3.17E-05	2.08E-01	1.85E-01
cg01513913	14	14q32.33	−4.72E-03	−1.55E-03	3.25E-05	5.20E-01	−1.09E-01
cg23594345	14	14q32.33	−9.68E-03	−1.49E-04	6.14E-05	9.73E-01	−1.99E-01
cg01208318	14	14q32.33	−8.63E-03	−9.63E-04	4.21E-05	8.27E-01	−2.18E-01
cg23161492	15	*ANPEP*	−7.55E-03	−9.10E-03	4.46E-05	3.95E-02	−2.73E-01
cg13500388	16	*CBFB*	−6.27E-03	−2.77E-03	6.18E-06	2.16E-01	−1.16E-01
cg10062919	17	*RARA*	−3.41E-03	−1.41E-03	1.84E-05	3.15E-01	−8.87E-02
cg00968616	17	*CUEDC1*	4.60E-03	−1.90E-04	2.56E-05	9.10E-01	7.15E-02
cg03636183	19	*F2RL3*	−9.68E-03	−7.95E-03	1.80E-06	1.18E-01	−6.63E-01
cg15159987	19	*CPAMD8*	−5.66E-03	2.20E-03	3.70E-06	2.97E-01	−1.66E-01
cg21473814	19	*CRTC1*	7.35E-03	4.00E-03	3.83E-05	2.16E-01	1.93E-01
cg11554391^a^	5	*AHRR*	−5.25E-03	−1.58E-02	9.14E-03	2.13E-05	−2.20E-01

CHR: chromosome of the CpG site; EWAS: epigenome-wide association analysis

^a^statistically significant for duration of smoking

Legend: Intensity of smoking is given in average number of cigarettes per day, and duration of smoking is length of smoking habit in years. The EWAS coefficient presented represents the methylation difference between smokers and never smokers at baseline, based on only the baseline information. It is presented to show that for all significant coefficients, the effect directions of increased intensity or increased duration are consistent with the effect of smoking vs not smoking

## Discussion

We have used longitudinal data with repeated measures of DNA methylation to examine changes in methylation over time associated with quitting or continuing smoking.

For the smoking cessation analysis, site-specific methylation changed the greatest (relative to changes observed for NS) for people who quit within 20 years prior to the follow-up examination. A total of 52 CpG sites were significant in our primary analysis of FS vs NS, with 32 significant for individuals quitting within (>0)-4 years and 30 significant for those who quit within 5–14 years prior to the follow-up examination (10 shared between them). Only one site showed a significant difference for individuals who had quit more than 14 years prior to the follow-up assessment.

Of the 52 significant sites, there were many from the same chromosome regions or genes. *AHRR* (8 sites), the aryl hydrocarbon repressor gene, located on chromosome 5, is involved in mediation of dioxin toxicity and functions also in cell regulation and growth. It is theorized that altered *AHRR* expression may have a deleterious effect on the body’s ability to eliminate environmental chemicals which may act as carcinogens [7]. *MYO1G* (3 sites), or unconventional myosin IG, is a plasma-membrane associated class 1 myosin and is found in abundance in lymphocytes [45]. It has been found to be related to cell death and a potential factor in cancer [46]. *HIVEP3* (2 sites) was found to be the gene with the most strongly associated CpG site (by p-value) in the meta-analysis of Joehanes et al. [12], where they note its role in bone formation. *GFI1* (Growth Factor Independent 1 Transcriptional Repressor) (4 sites) is a protein-coding gene, and its methylation was found to be the most robust mediator of the association between maternal smoking and birthweight in a recent study of children and newborns exposed to maternal and paternal smoking [47]. *ZNF668* (2 sites) is a zinc finger protein whose role in DNA repair, cell proliferation and cancer has been investigated [48]. Chromosome region 1p36 (2 sites) has been investigated as a region containing a possible tumor suppressor [49]; with investigations including the effects of methylation [50]. Methylation of regions on chromosome 2, 2p25 (2 sites) and 2q37 (3 sites), has recently been found to be associated with all-cause mortality [51], as was methylation of many of the other genes significant in this analysis, including *F2RL3*, *AHRR*, and region 14q32. *LRP5* (2 sites) plays a role in the Wnt signaling pathway, which influences bone formation and is a factor in skeletal disorders [52].

A site that is significant longitudinally is properly interpreted as one that is rapidly changing compared to within NS. Non-significant change does not necessarily imply that the methylation of FS has recovered to the level of NS. If indeed there is no significant change, but the methylation levels themselves remain different, it may indicate that the levels are only very slowly returning to those of NS, or not at all. Power considerations are also important: those sites that were significantly different between CS and NS for the EWAS may never show significant change in the TSQ analysis due to much smaller sample size for each category, a model that has fewer degrees of freedom and a smaller effect size. This does not imply that the methylation of these sites remains different to that of NS. This is most apparent for the difference between TSQ_L_ categories 1 and 2, where the difference between the significant cross-sectional and longitudinal coefficients for TSQ_L_ category 1 (264 and 32, respectively, a difference of 232) is much larger than the number of cross-sectionally significant sites for TSQ_L_ category 2 (104).

The sites that are not significant up to 4 years TSQ but are for between 5 and 14 years are difficult to interpret due to the implications of our categorization. It is possible the sites maintain the methylation levels of CS for years before rapidly changing later, a cascading effect in which certain methylation changes occur prior to others. Further possible explanations are attributable to the fact that the individuals with TSQ up to 4 years (TSQ_L_ category 1) are those who quit smoking between baseline and follow-up. Firstly, we do not have their methylation levels at cessation, only from some time prior, at baseline, at which point they were CS. Had their continued smoking to the time of cessation further driven their methylation levels and those of NS apart, the longitudinal effects seen would be diluted. Secondly, for other TSQ_L_ categories, we are examining the change in methylation for on average 7 years of non-smoking. Even considering a theoretically more rapid yearly change for individuals of TSQ_L_ category 1, the total effects measured (i.e., the regression coefficients) are based on at most 4 years of non-smoking. This change could thus be smaller in magnitude than a smaller per year change totaled over 7 years of non-smoking. Future studies could measure methylation directly before cessation to avoid these consequences.

The finer scale examination of reversion using medians of methylation change seems to indicate that methylation remains in general more dynamic for FS than NS even up to 40 years after cessation. Figure 2 shows in detail greater fluctuating methylation levels amongst FS than NS: after a certain length of time after quitting, methylation levels are not necessarily moving in opposite direction to the general effects of smoking, and in general changes remain stronger in magnitude than for NS. These indications, which warrant further investigation, seem to concur with Philibert et al. [16] that there can be overcorrection of methylation after cessation of smoking.

Regarding overall rates of reversion of disturbed methylation over time, it is of debate as to whether the relative persistence of differential methylation following cessation for certain CpG sites is due to slower reversion rates, or comparable reversion rates but a larger initial disturbance in methylation [8, 20]. Our results indicate the rate at which the methylation levels change in FS is related to the difference in methylation to NS, implying perhaps a type of exponential decay in the difference in methylation after cessation. Further investigation could investigate if this would be an appropriate model for some CpG sites, and if so, to determine a decay rate of the difference. Of particular interest are those sites with the largest absolute cross-sectional coefficient to longitudinal TSQ_L_ coefficient ratios. The implication of such a ratio being large is that these sites display an initially large difference between FS and NS, and this difference decays more slowly, relative to other sites. Of the 10 sites with the largest ratios in our study, 8 have been found to be “persistently differentially methylated” amongst FS in a previous study [20]. CpG sites which remain differentially methylated for long periods after cessation can aid us in understanding the course of smoking-related diseases – and thus the ongoing increased risk faced by FS – and act as biomarkers for past smoking exposure. Other markers, such as cotinine, a metabolite of nicotine, have short half-lives and their usefulness is thus limited [53]. Past candidates for CpG biomarkers of smoking include those at *AHRR* [11, 54], *F2RL3* [21] and the position 2q37 [11]. Our study confirms their utility in this regard, as our longitudinal results indicate that the methylation difference is large enough that reversion can still be measured significantly many years after cessation. Further, if multiple methylation measures per individual are available, both the levels and change could be combined in a biomarker to provide more accurate estimates of smoking history and risk of disease.

In terms of future avenues of investigation with regard to disease, additional further analysis could focus on the biology pertaining to those sites persistently differentially methylated, or even those sites with greater fluctuation in methylation, and the implications in disease etiology. We have presented here an array of diseases associated with both the significant loci and with smoking, but did not investigate incidence of these conditions in our population.

The investigation into continued smoking reveals that there are complex mechanisms behind the methylation levels within the blood. The results that indicate the effects are not consistently positively correlated with the effects of smoking in general – as seen through the significant longitudinal effects not necessarily showing the same direction with the cross-sectional effects – may be surprising considering that methylation of some sites has been found to be associated with cumulative smoking exposure (often given as “pack-years”, the product of average intensity of smoking and duration of smoking) [9, 18, 21]. Although the decrease in smoking intensity may partially explain the opposing longitudinal and cross-sectional effects, we see no conclusive evidence. Another explanation may lie in the fact that since our cohorts are composed of mostly older individuals, most have been smoking for many years (mean duration of smoking at baseline: 31.4 years). They may have reached or are approaching a methylation “peak” and changes at this stage may be too small to notice. Zhang et al. [21] show that for selected CpG sites annotated to the gene *F2RL3* the methylation response to dose of pack-years flattens after a certain level is achieved: the methylation effect is saturated. If such a model is accurate, and since many of the individuals in our sample are long-time smokers, longitudinal effects may be incorporating many individuals already at this stage, thus potentially “diluting” the contributions from earlier stage smokers. Indeed, since a CpG site does indeed have minimum and maximum methylation (0% - 100%) the effects cannot continue to compound across time after a certain point. The lack of significance results for duration (1 site), intensity (32 sites) and pack-years (we found 66 CpG sites of the 590 to be significant for pack-years, results not shown) may indicate a lack of power, a complex mechanism not addressed by our simple model, or a true lack of effect; this last possibility indicates that presence/absence of smoking habit (beyond a certain cut-off) may be the only relevant factor for certain CpG sites. A study of very early stage smokers may help to address this question. This lack of “new” smokers – either those that have just begun to smoke, or those with a very short history of smoking – is a weakness of this study, as we cannot examine the longitudinal effects within the early years of a smoking habit. These “new” smokers would likely be more informative than our long-term smokers on progression of longitudinal change in methylation. A further possibility for the seemingly conflicting results is interaction with other molecular factors. Recent studies [55, 56] have indicated that single-nucleotide polymorphisms influence the smoking-associated CpG sites. These studies further highlight the complexity of molecular networks, and underline the need for functional analysis.

The study has further limitations. Although longitudinal, only two time points were used, separated by approximately seven years. It is thus difficult to capture the shorter term, i.e. within the first few months, or longer term, i.e. decades, longitudinal effects of quitting smoking. Further, smoking studies often suffer from under-reporting, and a lack of data on consistency of smoking habits, particularly with regard to smokers attempting to quit.

Another weakness of the study is a type of selection bias. By the nature of the study – two time-points featuring identical individuals – we are likely excluding individuals most strongly affected by smoking: those that would have died, perhaps due to smoking-related illnesses, in the years between baseline and follow-up.

Finally, the lack of independent replication data weakens the reliability and generalizability of the results. It should be noted, however, that all CpG sites identified in this study as showing dynamism associated with continued smoking or with past smoking (TSQ analysis) were identified as cross-sectionally associated with smoking behavior in the extensive meta-analysis of Joehanes et al. [12], thus providing additional confidence in the results.

Strengths of the study include the longitudinal data – as mentioned, there is a scarcity of multiple time-point methylation data – and a relatively large sample size. The inclusion of well-documented covariates from the extensive KORA study lessens the possibility we are seeing confounded results.

## Conclusions

Our results provide insights into the rates of reversion of smoking-disturbed methylation levels and their continued fluctuation upon cessation of smoking. The results indicate that the most rapid reversion of methylation occurs within the first two decades following cessation of smoking, but that levels continue to fluctuate more for former smokers than for never smokers even beyond 30 years after cessation. Rates of reversion are related to the initial disturbance of methylation, with greater disturbance showing greater change across time. Site-specific results, including those for the previously identified genes *AHRR*, *F2RL3*, *GFI1*, and *MYO1G*, and chromosome regions 1p36, 2p25 and 2q37, have potential implications for both biomarkers and the treatment of human disease. We note that before-and-after studies on the short-term effects of smoking cessation would be beneficial to fuller understanding. We also demonstrate that the effects of continued smoking on methylation are complex, where duration and intensity of smoking habit and range of possible methylation all play interconnected roles.

## Additional files

Additional file 1: Table S1. Category descriptions for the time since quitting smoking analysis (TSQ_L_ categories). (XLS 24 kb)

Additional file 2: Table S2. Statistically significant results of the baseline epigenome-wide association study (EWAS). Presented are all CpG sites significant at a Bonferroni-corrected threshold of *P* < 0.05/449102 ≈ 1.1e-7 (for a family-wise type I error rate of 0.05). The coefficient represents the methylation difference in current smokers compared to never smokers. (XLS 112 kb)

Additional file 3: Figure S1. Longitudinal regression coefficients for each CpG site under investigation longitudinally, TSQ_L_ categories 4–7. The four panels display the coefficients and coefficient *p*-values for TSQ_L_ categories 4 through 7, respectively, for each CpG site under investigation. The longitudinal regression coefficients represent the rate of change of methylation M-value per year relative to never smokers. The results for TSQ_L_ categories 1 through 3 are given in Fig. 1. *Statistically significant: the longitudinal coefficient P falls below the Bonferroni-corrected threshold of 8.47e-5. (TIFF 84 kb)

Additional file 4: Table S3. Results for the longitudinal time since quitting analysis. The first sheet: Presents the sites statistically significant for TSQ_L_ category 1 or TSQ_L_ category 2. The columns “Sign. for TSQ_L_ category 1” and “Sign. for TSQ_L_ category 2” indicate whether the CpG site is significant longitudinally (reference category individuals who have never smoked) for TSQ_L_ category 1 (>0–4 years since quitting smoking at the time of the follow-up) and for TSQ_L_ category 2 (5–14 years). In addition to the longitudinal coefficients and *p*-values (representing the rate of change of methylation between baseline and follow-up per year relative to never smokers), cross-sectional coefficients are also given (representing the methylation difference to never smokers at baseline). “Chromosome” is the chromosome of the CpG site and “Gene or region” is based on the annotation file provided by Illumina (HumanMethylation450 v1.2 Manifest File). The remaining sheets: Results for all 590 CpG sites for each TSQ_L_ category and for the time coefficient. (XLS 695 kb)

Additional file 5: Table S4. Previously examined associations with disease for CpG sites and genes found significant in the time since quitting analysis. Presented are the references for human diseases/conditions for which i) an association has been found with smoking and ii) an association has been found with a CpG site (or its gene or region) that was found significant in the longitudinal time since quitting analysis in this study. (XLS 59 kb)

Additional file 6: Table S5. Gene overrepresentation analysis results based on the WebGestalt platform (webgestalt.org). For each TSQ_L_ category we extracted the CpG sites that showed nominal significance (*p* < 0.05) for that category or any later category and ran the WebGestalt overrepresentation enrichment analysis (based on the GLAD4U disease functional database, the default parameters (5–2000 genes per category, Benjamini-Hochberg multiple-testing correction) and the reference set “illumina_human_methylation_450”). Displayed are the top 10 diseases based on p-value. C: the number of reference genes in the category. O: the number of genes in the used gene list and also in the category. E: the expected number in the category. R: ratio of enrichment. PValue: *P*-value from the hypergeometric distribution. FDR: FDR from the Benjamini-Hochberg adjustment (XLS 44 kb)

Additional file 7: Figure S2. Heat map of median changes in methylation beta value from baseline to follow-up for current smokers, former smokers and never smokers. The color indicates direction of change in relation to the effect of smoking as found in the epigenome-wide analysis: red is the same direction, blue is opposite. Presented are all CpG sites under investigation. Figure 2 presents only those CpG sites with a median absolute change greater than 0.025 in at least one smoking category. (TIFF 438 kb)

Additional file 8: Figure S3. Change in methylation beta values from baseline to follow-up for current smokers, former smokers and never smokers, original TSQ_L_ categories. Upper panel: gives the length of the interquartile range over all CpG sites of the median change in methylation. A larger interquartile range indicates greater fluctuation in methylation between baseline and follow-up over the 590 CpG sites. Lower panel: gives the proportion of sites with consistent direction of change to the effect of smoking as found in the epigenome-wide analysis, “consistent” defined here as opposite in sign to the baseline effect of smoking. For both panels, the red line indicates the value for the never smoking individuals, the blue line is the smoothing loess curve as defined by the stat_smooth function with default values from the R package ggplot2, and the gray band is its 95% confidence interval. (TIFF 89 kb)

Additional file 9: Table S6. Spearman correlations between longitudinal coefficients and cross-sectional coefficients for each time since quitting smoking category. The longitudinal coefficients represent the rate of change of methylation M-value per year between baseline and follow-up relative to never smokers. The cross-sectional coefficients represent the methylation difference to never smokers at baseline. The correlations and *p*-values are given when using only the sites that are longitudinally significant for that smoking category, when using sites that are longitudinally significant for any smoking category, and when using all 590 investigated sites. (XLS 33 kb)

Additional file 10: Table S7. All results of the analysis of longitudinal effect of continued smoking. The longitudinal regression coefficients represent the rate of change of methylation M-value per year from baseline to follow-up for continued smokers relative to never smokers. The cross-sectional coefficients represent the methylation M-value difference to never smokers at baseline. Also presented are the results for the time coefficient from the model. “Chromosome” is the chromosome of the CpG site and “Gene or region” is based on the annotation file provided by Illumina (HumanMethylation450 v1.2 Manifest File). (XLS 179 kb)

Additional file 11: Figure S4. Boxplots of the methylation values for the CS-CS individuals (current smokers at both baseline and follow-up) and the NS-NS individuals (never smokers at both baseline and follow-up). Figure S4a shows the technically adjusted beta values and Figure S4b shows the methylation beta values after residualization to account for confounding (see Statistical Methods). (PDF 23 kb)

Additional file 12: Table S8. All results for the model incorporating both intensity and duration of smoking. Intensity of smoking is given in average number of cigarettes per day, and duration of smoking is length of smoking habit in years. The EWAS coefficient presented represents the methylation difference between smokers and never smokers at baseline, based on only the baseline information. It is presented to show whether the effect directions of increased intensity or increased duration are consistent with the effect of smoking vs not smoking. “Chromosome” is the chromosome of the CpG site and “Gene or region” is based on the annotation file provided by Illumina (HumanMethylation450 v1.2 Manifest File). (XLS 132 kb)

## Abbreviations

CpG: A cytosine-guanine dinucleotide locus in the DNA
CS: Current smokers
CS-CS: Current smokers at baseline (S4) who remained current smokers at follow-up (F4)
CS-FS: Current smokers at baseline (S4) who were former smokers at follow-up (F4)
DNA: Deoxyribonucleic acid
EWAS: Epigenome-wide association study
F4: The follow-up study (conducted between 2006 and 2008) of the S4 baseline study (conducted between 1999 and 2001)
FS: Former smokers
FS-FS: Former smokers at baseline (S4) who remained former smokers at follow-up (F4)
KORA: Kooperative Gesundheitsforschung in der Region Augsburg (Cooperative Health Research in the Augsburg Region)
NS: Never smokers
NS-NS: Never smokers at baseline (S4) who remained never smokers at follow-up (F4)
S4: Baseline study (conducted between 1999 and 2001) which was followed-up in the F4 follow-up study (conducted between 2006 and 2008)
SNP: Single nucleotide polymorphism
TSQ: Time since quitting smoking, measured in years
TSQ_L_: Time since quitting smoking, categorical variable as given in Additional file 1: Table S1
