# Supplementary figures and images for: The dynamics of smoking-related disturbed methylation: a two time-point study of methylation change in smokers, non-smokers and former smokers
Source: BMC Genomics. 2017 Oct 18;18:805. doi: 10.1186/s12864-017-4198-0 (PMC6389045; doi:10.1186/s12864-017-4198-0)

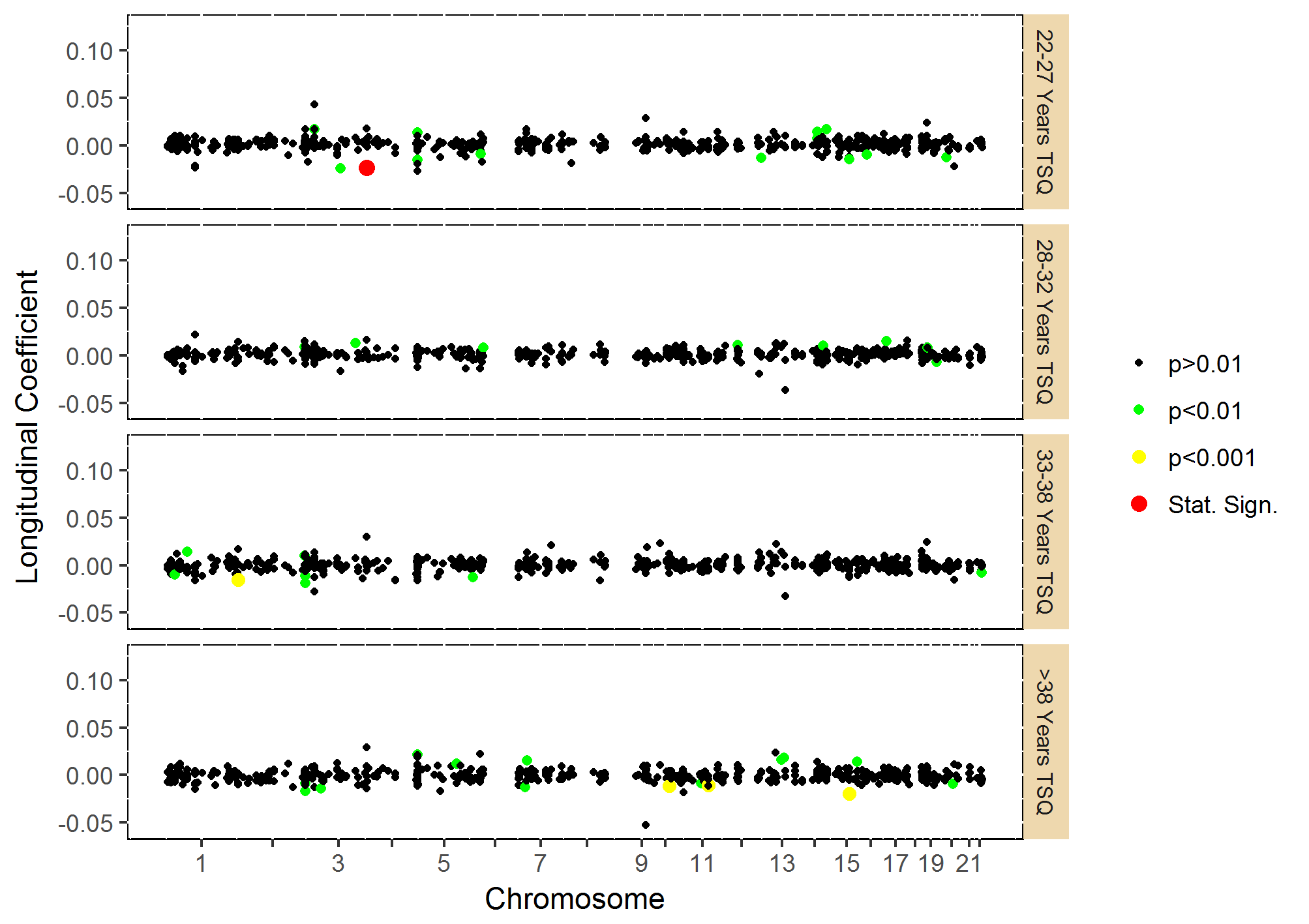

Supplement: Supplementary file 3 — Longitudinal regression coefficients for each CpG site under investigation longitudinally, TSQL categories 4–7. The four panels display the coefficients and coefficient p-values for TSQL categories 4 through 7, respectively, for each CpG site under investigation. The longitudinal regression coefficients represent the rate of change of methylation M-value per year relative to never smokers. The results for TSQL categories 1 through 3 are given in Fig. 1. *Statistically significant: the longitudinal coefficient P falls below the Bonferroni-corrected threshold of 8.47e-5. (TIFF 84 kb) [file 12864_2017_4198_MOESM3_ESM.tiff]

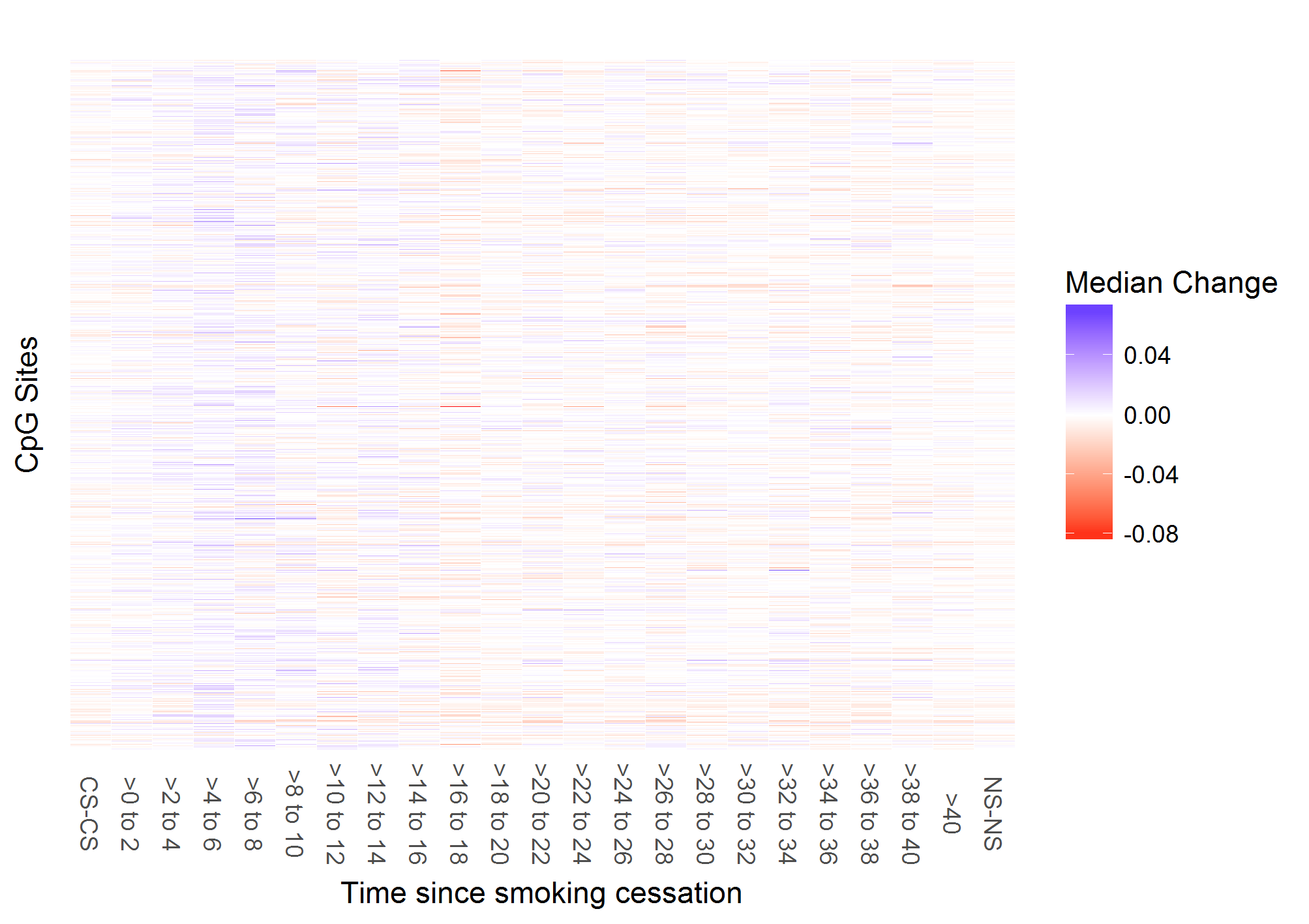

Supplement: Supplementary file 7 — Heat map of median changes in methylation beta value from baseline to follow-up for current smokers, former smokers and never smokers. The color indicates direction of change in relation to the effect of smoking as found in the epigenome-wide analysis: red is the same direction, blue is opposite. Presented are all CpG sites under investigation. Figure 2 presents only those CpG sites with a median absolute change greater than 0.025 in at least one smoking category. (TIFF 438 kb) [file 12864_2017_4198_MOESM7_ESM.tiff]

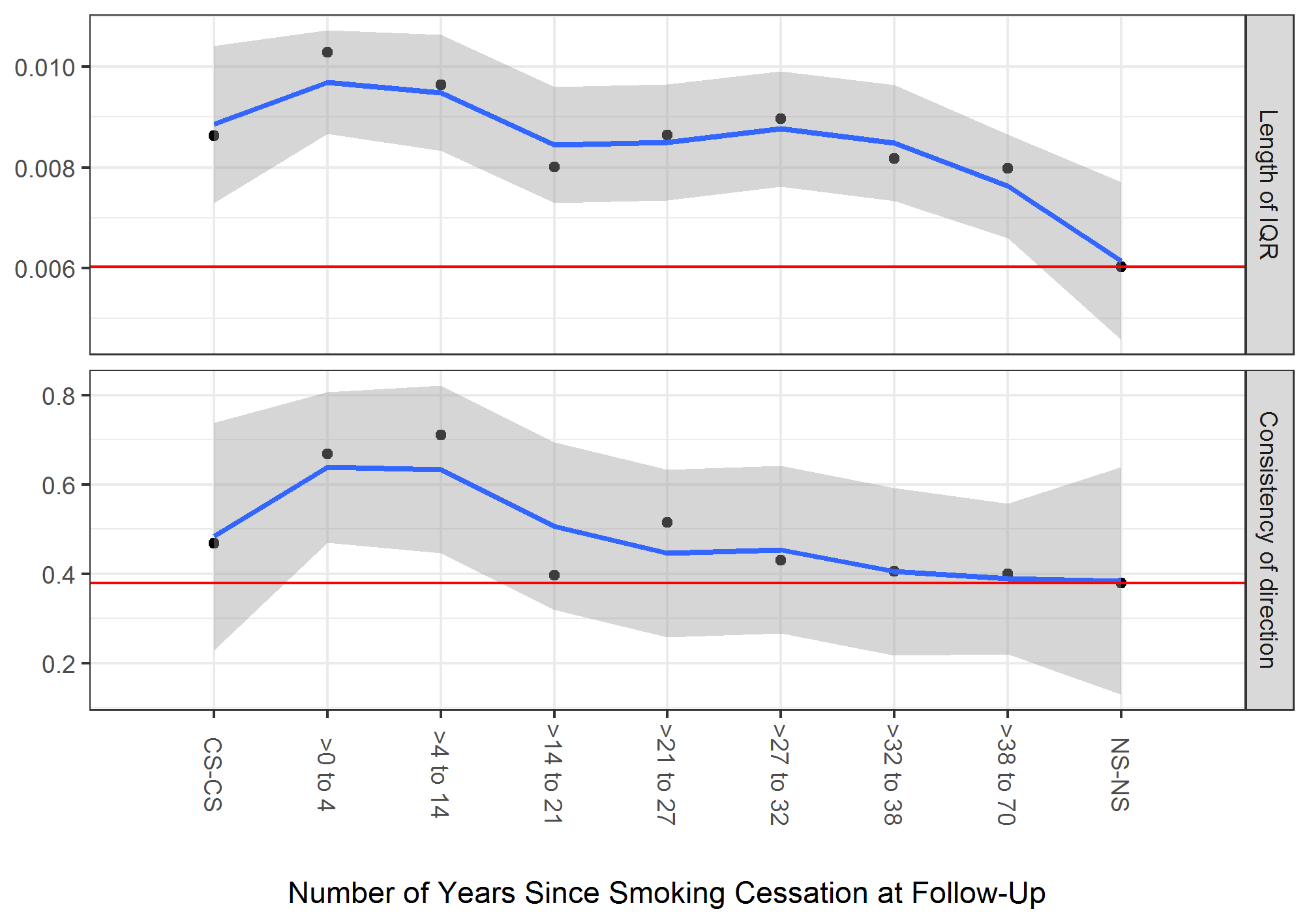

Supplement: Supplementary file 8 — Change in methylation beta values from baseline to follow-up for current smokers, former smokers and never smokers, original TSQL categories. Upper panel: gives the length of the interquartile range over all CpG sites of the median change in methylation. A larger interquartile range indicates greater fluctuation in methylation between baseline and follow-up over the 590 CpG sites. Lower panel: gives the proportion of sites with consistent direction of change to the effect of smoking as found in the epigenome-wide analysis, “consistent” defined here as opposite in sign to the baseline effect of smoking. For both panels, the red line indicates the value for the never smoking individuals, the blue line is the smoothing loess curve as defined by the stat_smooth function with default values from the R package ggplot2, and the gray band is its 95% confidence interval. (TIFF 89 kb) [file 12864_2017_4198_MOESM8_ESM.tiff]
