# Supplementary material for: The dynamics of smoking-related disturbed methylation: a two time-point study of methylation change in smokers, non-smokers and former smokers
Source: BMC Genomics. 2017 Oct 18;18:805. doi: 10.1186/s12864-017-4198-0 (PMC6389045; doi:10.1186/s12864-017-4198-0)
Supplement: Supplementary file 11 — Boxplots of the methylation values for the CS-CS individuals (current smokers at both baseline and follow-up) and the NS-NS individuals (never smokers at both baseline and follow-up). Figure S4a shows the technically adjusted beta values and Figure S4b shows the methylation beta values after residualization to account for confounding (see Statistical Methods). (PDF 23 kb) [file 12864_2017_4198_MOESM11_ESM.pdf]

Figure S4a

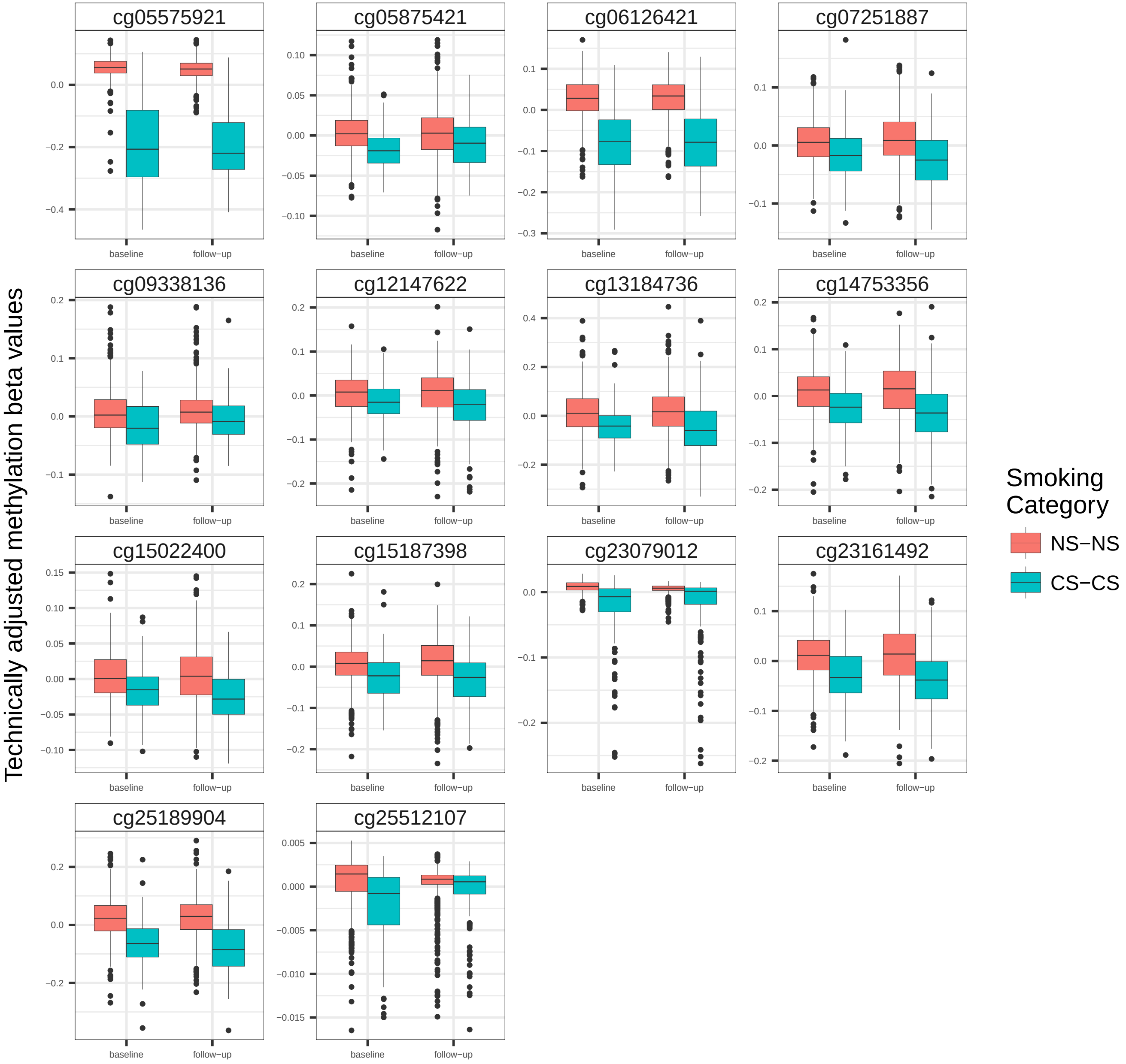
cg05575921
cg05875421
cg06126421
cg07251887
cg09338136
cg12147622
cg13184736
cg14753356
cg15022400
cg15187398
cg23079012
cg23161492
cg25189904
cg25512107
baseline
follow−up
Technically adjusted methylation beta values
Smoking Category
NS−NS
CS−CS

Figure S4b

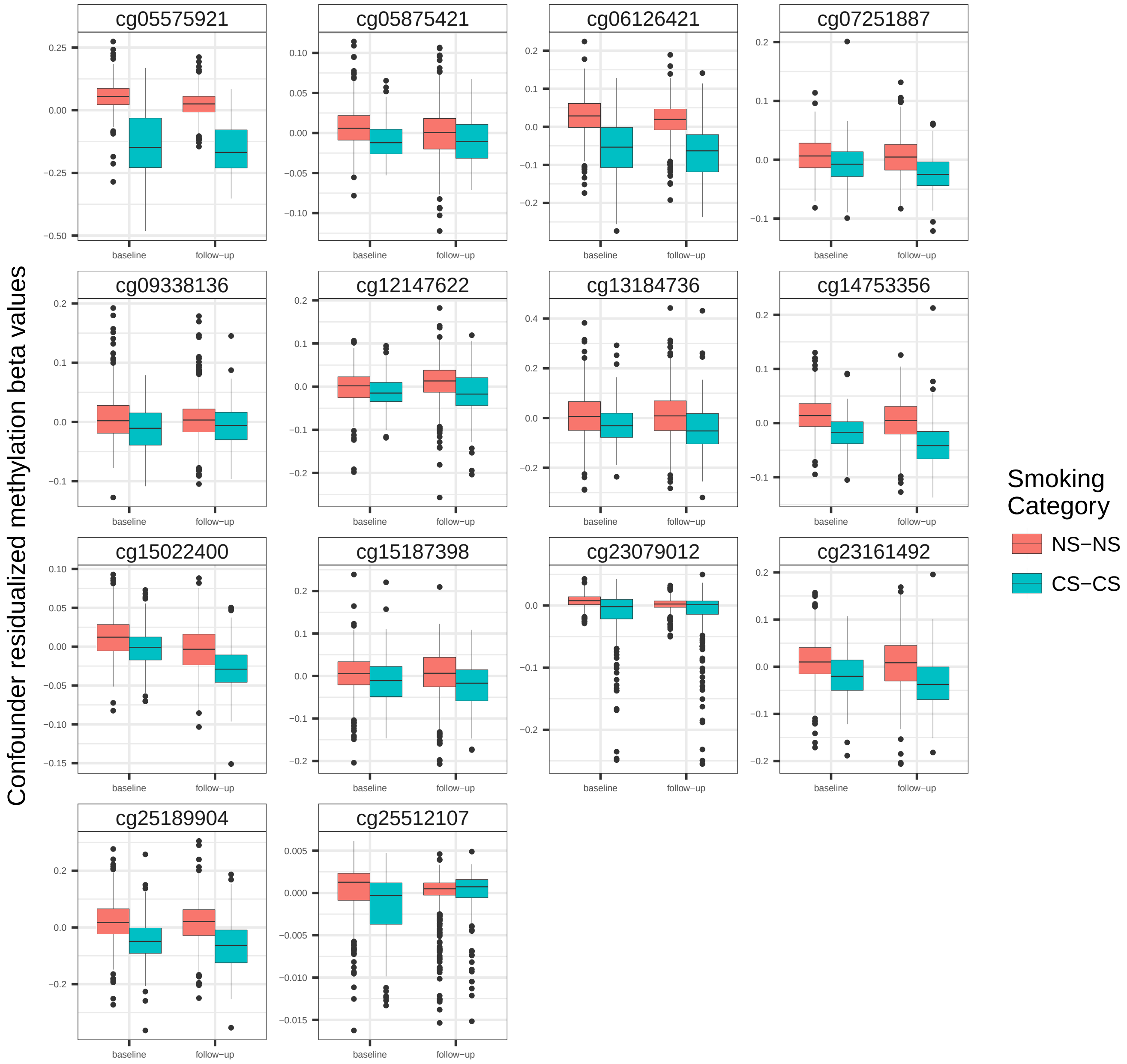

cg05575921
cg05875421
cg06126421
cg07251887
cg09338136
cg12147622
cg13184736
cg14753356
cg15022400
cg15187398
cg23079012
cg23161492
cg25189904
cg25512107
baseline
follow−up
Confounder residualized methylation beta values
Smoking Category
NS−NS
CS−CS
